# Synaptonemal Complex-Deficient *Drosophila melanogaster* Females Exhibit Rare DSB Repair Events, Recurrent Copy-Number Variation, and an Increased Rate of *de Novo* Transposable Element Movement

**DOI:** 10.1534/g3.119.400853

**Published:** 2019-12-27

**Authors:** Danny E. Miller

**Affiliations:** * Division of Medical Genetics, Department of Medicine, and; † Division of Genetic Medicine, Department of Pediatrics, University of Washington, Seattle, Washington 98105, and; ‡ Seattle Children’s Hospital, Seattle, Washington 98105

**Keywords:** meiosis, whole-genome sequencing, crossing over, noncrossover gene conversion, c(3)G, corolla, synaptonemal complex, transposable element, copy-number variation, sister chromatid exchange

## Abstract

Genetic stability depends on the maintenance of a variety of chromosome structures and the precise repair of DNA breaks. During meiosis, programmed double-strand breaks (DSBs) made in prophase I are normally repaired as gene conversions or crossovers. DSBs can also be made by other mechanisms, such as the movement of transposable elements (TEs), which must also be resolved. Incorrect repair of these DNA lesions can lead to mutations, copy-number changes, translocations, and/or aneuploid gametes. In *Drosophila melanogaster*, as in most organisms, meiotic DSB repair occurs in the presence of a rapidly evolving multiprotein structure called the synaptonemal complex (SC). Here, whole-genome sequencing is used to investigate the fate of meiotic DSBs in *D. melanogaster* mutant females lacking functional SC, to assay for *de novo* CNV formation, and to examine the role of the SC in transposable element movement in flies. The data indicate that, in the absence of SC, copy-number variation still occurs and meiotic DSB repair by gene conversion occurs infrequently. Remarkably, an 856-kilobase *de novo* CNV was observed in two unrelated individuals of different genetic backgrounds and was identical to a CNV recovered in a previous wild-type study, suggesting that recurrent formation of large CNVs occurs in Drosophila. In addition, the rate of novel TE insertion was markedly higher than wild type in one of two SC mutants tested, suggesting that SC proteins may contribute to the regulation of TE movement and insertion in the genome. Overall, this study provides novel insight into the role that the SC plays in genome stability and provides clues as to why the sequence, but not structure, of SC proteins is rapidly evolving.

Programmed double-stranded DNA breaks (DSBs) made during prophase of meiosis I are a critical step in the formation of healthy gametes, yet they are potentially catastrophic events for cells. The meiotic break repair machinery must therefore accurately resolve DSBs as either crossovers (COs) or noncrossover gene conversions (NCOGCs). Because more DSBs are made than will be repaired as COs, the majority of DSBs are repaired as NCOGCs, which are nonreciprocal exchange events that result in the 3:1 segregation of alleles.

Crossing over is essential to ensure the proper segregation of homologous chromosomes during subsequent meiotic divisions. Crossovers occur within the context of a large, multiprotein structure called the synaptonemal complex (SC), which forms between homologous chromosomes. In most organisms, DSBs must be made before SC formation can occur, and functional SC is required for proper DSB repair (de Massy 2012; Zickler and Kleckner 2015). However, in *Drosophila melanogaster*, the SC is necessary for both robust DSB formation and DSB repair (Lake and Hawley 2012); in the absence of functional SC, DSBs are made at about 20–40% of the wild-type level (Mehrotra and McKim 2006; Collins *et al.* 2014).

The Drosophila SC protein C(3)G is functionally homologous to the transverse filament proteins SYCP-1 in mammals and ZIP1 in budding yeast (Page and Hawley 2001). While females heterozygous for a loss-of-function *c(3)G* allele appear to build normal SC, homozygous females do not build SC and are thus unable to resolve into COs those DSBs that do occur (Page and Hawley 2001). A previous study examining NCOGC events at a single locus in Drosophila recovered no events from *c(3)G* homozygous females but did not report the number of progeny scored (Carlson 1972), thus whether DSBs can be repaired as NCOGCs in females lacking functional SC is unknown. Like C(3)G, the Drosophila SC protein Corolla is also required for SC formation. Mutants in *corolla* exhibit phenotypes typical of Drosophila SC mutants, including a reduced number of DSBs (∼40% as assayed by γH2AV foci) and increased levels of chromosome segregation defects (Collins *et al.* 2014). As with *c(3)G* homozygous females, it is not known how DSBs are repaired in *corolla* homozygotes.

While it is evident the SC plays a vital role in resolving DSBs into COs, its role in other meiotic processes is less obvious. For example, there is some evidence for a link between SC formation and the position of transposable elements (TEs), but the data are not definitive (Pearlman *et al.* 1992; Hernández-Hernández *et al.* 2008; Marcon *et al.* 2008; van der Heijden and Bortvin 2009). Transposable elements are mobile genetic elements active during different stages of gametogenesis. They can be divided into two classes: Class 1, or retrotransposons, replicate using a copy-and-paste method to insert copies of themselves into new locations in the genome, while Class 2, or DNA transposons, use a cut-and-paste method to move from one position in the genome to another. While the tripartite structure of the SC is highly conserved among many species, the genes that make up the SC are evolving rapidly at the sequence level, making their identification even within a species group challenging (Fraune *et al.* 2012; Hemmer and Blumenstiel 2016). The reason for the rapid sequence evolution of the SC is unknown, but it could be to counter the effects of TE movement during meiosis. In Drosophila female meiosis, the rate at which TE movement occurs and whether the SC has any role in facilitating or limiting TE movement remains unclear.

In the current study, whole-genome sequencing was used to investigate individual meiotic events in male offspring from females heterozygous or homozygous for a loss-of-function allele of *c(3)G*. While the number and distribution of CO and NCOGC events in individuals from females heterozygous for *c(3)G* was similar to wild type, in progeny arising from *c(3)G* homozygous mothers (which do not build SC), no COs and only one likely NCOGC event were recovered. The recovery of a single presumed NCOGC event suggests that while repair of DSBs via NCOGC may be possible in females lacking functional SC, it is not a common mechanism of DSB repair. Consistent with the high levels of chromosome missegregation observed in SC mutants, *X0* males lacking a *Y* chromosome, males with *4^th^* chromosome gain or loss, and flies that appeared phenotypically male were also recovered.

These data also provide information on what role, if any, the SC components C(3)G and Corolla play in facilitating or inhibiting TE movement during meiosis. In the current study of SC-defective mutants, novel TE insertions were curiously significantly elevated in *c(3)G* homozygotes but similar to wild type in *corolla* homozygotes. Shared and novel large-scale TE-mediated CNVs were also identified in progeny from all genotypes. Remarkably, one of these CNVs was observed in three unrelated individuals—two from this study and one from a separate study of individual meiotic events in wild type (Miller *et al.* 2016b)—suggesting that, similar to humans (Itsara *et al.* 2009), recurrent CNVs may be a common occurrence in Drosophila. Overall, this work helps further our understanding of how meiotic cells cope with DNA breaks and maintain genetic stability.

## Methods

### Fly Stocks and husbandry

The loss-of-function allele *c(3)G^68^* (Page and Hawley 2001) was crossed into stocks isogenic for either *w^1118^* or Canton-S strain polymorphisms (Miller *et al.* 2012). Females homozygous for Canton-S *X* and *2^nd^* chromosomes and heterozygous for the *c(3)G^68^* loss-of-function allele were crossed to *w^1118^* males to generate females heterozygous for Canton-S and *w^1118^* strain polymorphisms. These heterozygous females were then crossed again to isogenic *w^1118^* males and individual male progeny were isolated for sequencing (Figure S1). Females heterozygous for *w^1118^* and Canton-S *X* and *2^nd^* chromosomes and homozygous for *c(3)G^68^* were crossed to isogenic *w^1118^* males and individual male offspring were isolated for sequencing (Figure S1). Progeny from corolla^*129*^ homozygous females were generated by crossing virgin corolla^*129*^ females to sibling males and collecting both male and female progeny (Figure S1). All crosses were done using a single male and female, and females were allowed to lay eggs for 7 days before being removed from a vial. Male offspring used for sequencing were collected between days 12 and 15 post-fertilization. All flies were kept on standard cornmeal-molasses media and maintained at 25°.

### DNA preparation and sequencing

For all flies, DNA was prepared from single adults using the Qiagen DNeasy Blood & Tissue Kit. All flies were starved for 4 hr before freezing at -80° for at least 1 hr. One ng of DNA from each was fragmented to 250-bp fragments by adjusting the treatment time to 85 sec using a Covaris S220 sonicator (Covaris Inc.). Libraries were prepared using a Nextera DNA Sample Prep Kit and Bioo Scientific NEXTflex DNA Barcodes. The resulting libraries were purified using Agencourt AMPure XP system (Beckman Coulter) then quantified using a Bioanalyzer (Agilent Technologies) and a Qubit Fluorometer (Life Technologies). Samples from *c(3)G^68^* homozygous females were run on a HiSeq 2500 in rapid mode as either 100-bp paired-end or 125-bp paired-end samples using HiSeq Control Software 1.8.2 and Real-Time Analysis (RTA) version 1.17.21.3. Samples from the *c(3)G^68^* heterozygous and corolla^*129*^ homozygous experiments were run as 150-bp paired-end on a HiSeq 2500 in rapid mode using HiSeq Control Software 2.2.58 and RTA version 1.18.64. Secondary Analysis version CASAVA-1.8.2 was run to demultiplex reads and generate FASTQ files. Per-sample sequencing and alignment statistics can be found in Table S1.

### DNA alignment, SNP calling, and identification of CO and NCOGC events

Alignment to the Drosophila reference genome (dm6) was performed using bwa version 0.7.7-r441 using default parameters (Li and Durbin 2009). Single nucleotide and insertion or deletion polymorphisms were identified using SAMtools version 1.9 (Li *et al.* 2009). Candidate CO and NCOGC events were identified as described in Miller *et al.* (2016b).

### Depth-of-coverage calculations

Depth of coverage for each chromosome arm was calculated by summing the total read depth for each base position then dividing by the length of the entire chromosome arm. Because of the repetitive nature of the *Y* chromosome, analysis was limited to *chrY*:332,000–510,000 (Table S1).

### Validation of NCOGCs by PCR

Nine candidate NCOGC events were identified in 93 males from *c(3)G^68^* females and examined by PCR and Sanger sequencing; Phusion polymerase (NEB) was used according to the manufacturer’s instructions. Only one of the nine putative conversion events validated as real in male c3g-hom-6.4. All primers used can be found in Table S2.

### Calculation of expected NCOGC events

The number of NCOGCs expected to be recovered from 93 individuals from *c(3)G^68^* females if all DSBs on the *X* and *2^nd^* chromosomes were repaired as NCOGCs was estimated by performing 100,000 trials of randomly distributing an estimated number of DSBs among the *X* and *2^nd^* chromosomes using the SNP density of a *w^1118^*/Canton-S heterozygote. Given that DSBs in *c(3)G^68^* females are made at 20% of the wild-type rate of 18–20 DSBs per meiosis (Mehrotra and McKim 2006), a per-arm number of DSBs was estimated as 0–2 per meiosis. Each break was randomly assigned to a chromosome arm, then to a random chromatid. A random chromatid was then selected to be recovered. NCOGC tract length was assumed to be a minimum of 250 bp and a maximum of 1000 bp (Miller *et al.* 2016b). An NCOGC was predicted to be recoverable if the tract involved at least one high-quality SNP that differentiated the *w^1118^* and Canton-S genotypes. The estimate of the number of NCOGCs that should be recovered from individual offspring of *c(3)G^68^*/+ females was calculated by multiplying the wild-type per-arm NCOGC rate of 0.3 (Miller *et al.* 2016b) by 120, the number of arms studied.

### Identification of novel deletion polymorphisms

Novel deletions were identified using two approaches. Deletions smaller than 30 bp were identified using SAMtools (Li *et al.* 2009). For each class of progeny (wild type, *c(3)G^68^*, *c(3)G^68^*/+, and corolla^*129*^) a custom script identified any deletion, regardless of quality score, from all vcf files that did not overlap repetitive regions as defined by Repeatmasker (Smit *et al.*). Novel deletions were those with quality scores over 200 (as determined by SAMtools) that did not fall within 100 bp of another deletion on a different offspring. Candidate novel deletions were validated visually using IGV (Thorvaldsdóttir *et al.* 2013). Data for both wild type and *c(3)G^68^* were also analyzed using GATK HaplotypeCaller (McKenna *et al.* 2010), but no deletions not identified by SAMtools were found, thus the remainder of the analysis was completed with SAMtools. Larger deletions were identified using Pindel (Ye *et al.* 2009). For each class of progeny, Pindel was run using default settings with an average insert size of 200 bp. Output files for each class of progeny were analyzed as a group and candidate novel deletions were visually validated using IGV.

### Construction of synthetic genomes and sequencing reads

To determine what percentage of small or large *de novo* deletion polymorphisms would be identified by SAMtools and Pindel, synthetic genomes were computationally modified with deletions of varying sizes then analyzed using the approach described above. Two classes of genomes were generated: 100 with deletions 1–10 bp long, and 100 with deletions 1–1,000 bp long. For each individual, two genomes were generated: one with an *X* and without a *Y* chromosome, and one with a *Y* and without an *X*. For each of these genomes, a single nucleotide was randomly changed approximately once every 500 nucleotides to a randomly selected A, G, C, or T. Next, for each genome with an *X* and without a *Y* chromosome 2–6 DSBs (approximately 20% of the 18–20 DSBs expected in wild-type (Mehrotra and McKim 2006)) were randomly placed on one of four haplotypes in a euchromatic location in the genome. Each of these DSBs was randomly determined to have a deletion between either 1–10 bp or 1–1,000 bp beginning at the site of the DSB. One haplotype of these four was then randomly chosen as the genome for the individual. For each individual, ART was used to generate synthetic reads for both genomes with a read depth of approximately 10x (Huang *et al.* 2012). FASTQ files were then combined into a single forward and a single reverse file—and thus represented data from an *XY* individual with 20x depth of coverage—that were then aligned to the *D. melanogaster* reference genome as above. SNPs, insertion/deletion polymorphisms, and larger deletions were identified as described above with SAMtools (Li *et al.* 2009) and Pindel (Ye *et al.* 2009). Deletions generated per individual genome can be found in Table S3.

### Identification of transposable element insertions

To identify TE insertions, split and discordant read pairs were isolated from alignment files using SAMBLASTER (Faust and Hall 2014). BLAST (Altschul *et al.* 1997) was then used to annotate individual split or discordant reads using the *D. melanogaster* canonical TE set (Kaminker *et al.* 2002). Split and discordant clusters that contained more than five reads aligning to a specific TE family were considered candidate TE insertion sites. Novel insertions were detected by a custom script that compared insertions in one population or stock to related stocks or populations and were visually validated using IGV (Thorvaldsdóttir *et al.* 2013). Mosaic insertions on the *X* chromosome were identified visually using IGV.

### Identification of CNV events

CNV events were identified as described in Miller *et al.* (2016b). Briefly, average depth of coverage for each individual chromosome arm was determined, then the log_2_ depth of coverage for 5-kb nonoverlapping windows was calculated and plotted to reveal large regions of deletions or duplications.

### Data availability

Illumina data generated for this project are available at the National Center for Biotechnology Information (https://www.ncbi.nlm.nih.gov/). Data for individuals from *c(3)G^68^* females can be found under project PRJNA565835, data for males from *c(3)G^68^* heterozygous females are under project PRJNA565834, and data for males and females from corolla^*129*^ females are under project PRJNA565794. Wild-type data used in this study were obtained from project PRJNA307070 (Miller *et al.* 2016b). Original data underlying this manuscript can be accessed from the Stowers Original Data Repository at http://www.stowers.org/research/publications/libpb-1476. All code used in this project is available at GitHub (https://github.com/danrdanny/c3g-corolla-project/). Supplemental material available at figshare: https://doi.org/10.25387/g3.10006634.

## Results

### Analysis of individual meiotic events from c(3)G^68^ heterozygous and homozygous females

While in many organisms DSBs are made in the absence of SC (de Massy 2012; Zickler and Kleckner 2015), Drosophila is unique in that SC is required for robust DSB formation (Lake and Hawley 2012). *D. melanogaster* females homozygous for loss-of-function alleles of SC genes make DSBs at a rate approximately 20–40% that of wild type (Mehrotra and McKim 2006; Collins *et al.* 2014), and it remains unclear how these DSBs are repaired. Studies using visual markers in Drosophila have shown that repair of DSBs by crossing over is substantially reduced or completely abolished in females unable to construct full-length SC, and it is not known if these DSBs can be repaired by other pathways, such as NCOGC or nonhomologous end-joining (NHEJ) (Gowen 1933; Hall 1972; Page and Hawley 2001; Manheim and McKim 2003; Jeffress *et al.* 2007; Page *et al.* 2007; Collins *et al.* 2014).

To better understand this process, whole-genome sequencing was performed on individual male progeny from mothers heterozygous for wild-type Canton-S and *w^1118^ X* and *2^nd^* chromosomes and either homozygous or heterozygous for the loss-of-function allele *c(3)G^68^* on chromosome *3*. Male progeny from *c(3)G^68^* homozygous mothers (96 males from 10 females) represent the experimental group lacking SC and will hereafter be referred to as *c(3)G* offspring, and male progeny from *c(3)G^68^* heterozygous mothers (40 males from two females) represent the control group with functional SC and will be referred to as *c(3)G/+* offspring (Figure S1).

While Drosophila males normally have an *X* and a *Y* chromosome, sex is determined by the ratio of *X* chromosomes to autosomes rather than the presence of a *Y*, thus *X0* flies are male. This is seen when *X* chromosome missegregation (nondisjunction) leads to no maternal sex chromosome contribution, with a paternally inherited *X*. Triploid flies carrying three copies of each autosome and two *X* chromosomes may also be phenotypically male and are known as intersex males (Bridges 1921). To assay for *X* and *4^th^* chromosome nondisjunction and the presence of triploid flies, depth of coverage was calculated for each chromosome arm as a percentage of one of the autosomes (Table S1). Males carrying the expected number of *X* chromosomes should have *X* and *Y* chromosome depth of coverage half that of an autosome and *4^th^* chromosome depth of coverage equal to an autosome. As expected, all 40 male offspring from the *c(3)G/+* control group were diploid with an *X* and a *Y* chromosome as well as two copies of the *4^th^* chromosome. Meanwhile, among the offspring from the SC-deficient *c(3)G* experimental group, 25 were found to be *X0* males carrying an *X* chromosome with the *w^1118^* haplotype and six were found to have three *4^th^* chromosomes (Table S1, Figure S2). These 25 *X* chromosomes were most likely paternally inherited, since nondisjunction of the *X* or *Y* chromosome in wild-type Drosophila males has been shown to occur in fewer than 1 in 5,000 meioses (Boschi *et al.* 2006); however, because the mother was heterozygous for a *w^1118^* chromosome, there is a small, albeit unlikely, possibility that these *X* chromosomes were maternally derived. The high levels of nondisjunction in *c(3)G* homozygous females (42% *X* and 12% *4^th^*) was expected and is similar to previous work using the *c(3)G^68^* allele (39% *X* and 27% *4^th^*) (Hall 1972).

Three of the 96 *c(3)G* offspring had an *X* chromosome depth of coverage approximately 67% that of chromosomes *2* and *3*, with two of these three also carrying a *Y* chromosome (Figure S2, Table S1). Allele frequency for each SNP on each chromosome arm was calculated and revealed that all three were triploid, with one *XX:222:333* and two *XXY:222:333* offspring (Figure S3). The *XX:222:333* intersex offspring was also mosaic for loss of a *4^th^* chromosome, with 75% depth of coverage of the *4^th^* compared to chromosome *2L*, suggesting post-meiotic loss of the *4^th^* in *XX:222:333:444* cells (Figure S2). The recovery of intersex individuals was not surprising as previous studies have noted an increase in the number of triploid individuals recovered from *c(3)G* mutants (Gowen 1933; Lindsley and Zimm 1992). These three offspring were excluded from subsequent analysis.

**Figure 1:**
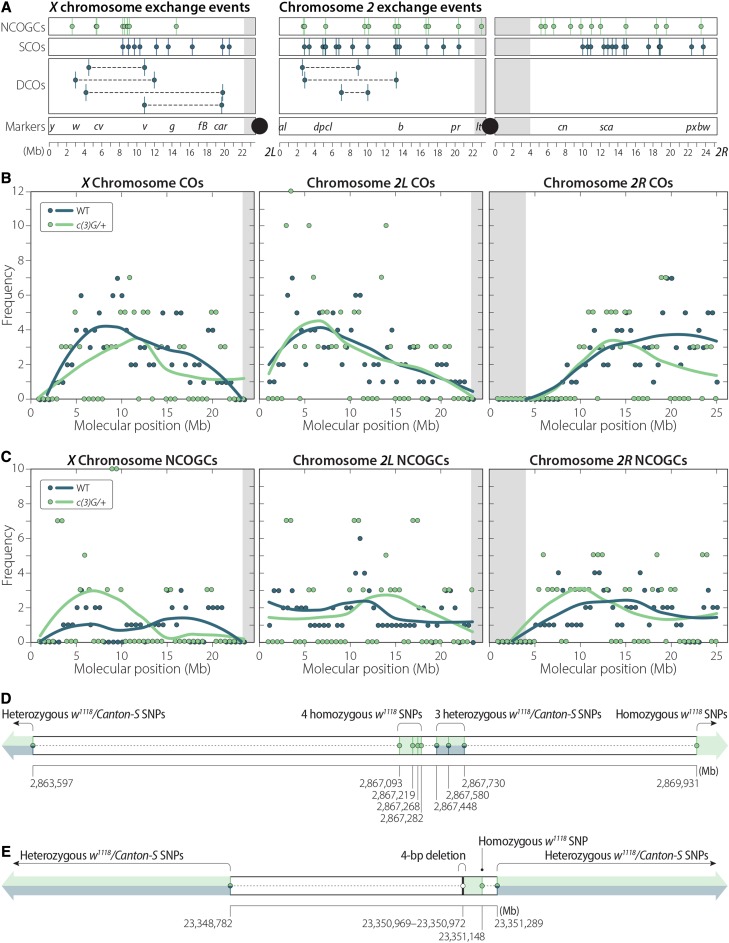
CO and NCOGC events recovered in this study (details are in Table S4 and Table S5). (A) Individual NCOGC, single crossover (SCO), and double crossover (DCO) events recovered per chromosome arm in *c(3)G^68^/+* females. No DCOs were recovered on *2R* in *c(3)G^68^/+*, and no COs of any type were recovered in *c(3)G^68^* homozygotes. Gray shading indicates heterochromatic regions; black circles represent centromeres. (B) Coefficient of exchange for all 55 CO events from *c(3)G^68^/+* females recovered in this study compared to wild-type data from Miller *et al.* (2016b). Points represent observed frequency; the line is a best-fit line through the data. (C) Coefficient of exchange for all 32 NCOGC events recovered from *c(3)G^68^/+* females compared to the same wild-type data as in (B). (D) Detail of the only CO-associated GC recovered in this study. The CO could have occurred at one of two positions on chromosome *2L*, either between SNPs at positions 2,863,597 and 2,867,093 with the CO-associated GC being the heterozygous tract between positions 2,867,448 and 2,867,730. Alternatively, the CO may have occurred between 2,867,730 and 2,869,931 with the CO-associated GC defined by the 4 SNPs between 2,867,093 and 2,867,282. No similar CO-associated GC events were recovered in a previous analysis of 196 individual meiotic events from wild-type females (Miller *et al.* 2016b) but have been reported in previous studies in Drosophila (Curtis *et al.* 1989). (E) Structure of the single NCOGC event recovered from a homozygous *c(3)G^68^* female in this study. This NCOGC, validated by PCR and Sanger Sequencing, was defined by a 4-bp deletion on one side and a SNP on the other, both from the *w^1118^* line. The NCOGC has a maximum possible tract length of 2,507 bp and a minimum tract length of 180 bp.

CO and NCOGC events were then identified on the *X* and *2^nd^* chromosomes in both *c(3)G* and *c(3)G*/+ male offspring through changes in polymorphisms in each fly. (CO and NCOGC events were not analyzed on the *3^rd^* because *c(3)G* lies on this chromosome, nor were they analyzed on the 25 paternally inherited *X* chromosomes carried by *X0 c(3)G* offspring.) A total of 41 single COs and 7 double COs were identified in *c(3)G*/+ offspring (Figure 1A, Table S4), with a frequency of exchange similar to previous observations in wild type for all three arms (Figure 1B). A total of 32 NCOGCs were also identified (Figure 1C, Table S5), not significantly different than the 36 expected to be recovered based on wild-type rates (Miller *et al.* 2016b). Previous work has shown rates of crossing over similar to wild type for the *c(3)G^68^* allele when heterozygous (Hall 1977), but higher rates of crossing over for *c(3)G^17^* as a heterozygote (Hinton 1966). While the *c(3)G^68^* allele is a known point mutation, the *c(3)G^17^* allele (also historically known as *c(3)G^1^*) is a TE insertion that disrupts the function of the gene (Page and Hawley 2001), and the reason for the difference in exchange between these two alleles is not clear.

Among 93 *c(3)G* homozygous offspring, no CO events were recovered on the *X* or *2^nd^* chromosomes, but a single NCOGC event in male c3g-hom-6.4 was identified and validated by PCR and Sanger sequencing. This event occurred on a chromosome with the Canton-S haplotype, which could have occurred only in the heterozygous *w^1118^*/Canton-S mother and thus was not contributed by the isogenic *w^1118^* father. This NCOGC was minimally defined by a 4-bp deletion on the 5′ side (*2R*:23,350,969–23,350,972, release 6 coordinates) and a single polymorphism on the 3′ side (*2R*:23,351,148) (Figure 1E, Table S5). Because it was defined by these two closely located polymorphisms that created two changes identical to the other haplotype used in this study, it is unlikely the event was the result of *de novo* somatic mutation. Moreover, the average depth of coverage within the 1-kb interval surrounding the two polymorphisms was 54x, similar to the average depth of coverage for chromosome *2R* for this individual, making it unlikely that this NCOGC was due to a deletion or duplication of this interval. Additionally, the minimum and maximum possible widths of this NCOGC are 180 bp and 2,507 bp, respectively, well within ranges observed in wild type (Hilliker *et al.* 1994; Miller *et al.* 2016b). Because homologous chromosomes pair prior to meiotic onset, this NCOGC could be the result of DSB repair in a pre-meiotic cell (Bosco 2012; Joyce *et al.* 2012). Unfortunately, there are no reliable estimates of the rate at which pre-meiotic NCOGC occurs, making the likelihood difficult to assess.

Females homozygous for *c(3)G* loss-of-function alleles make DSBs at ∼20% the level of wild type (Mehrotra and McKim 2006). To estimate the number of NCOGCs that should have been recovered in the *c(3)G* dataset if DSB repair as NCOGCs occurred frequently, a simulation was performed. This model randomly distributed DSBs among 68 *X* and 93 *2^nd^* chromosome arms as if they occurred at a rate 20% that of wild type. The model estimated that 37–62 NCOGCs should have been recovered if all DSBs that occurred were repaired as NCOGCs (since COs do not occur in *c(3)G^68^* homozygotes). The recovery of a single candidate NCOGC event is significantly less than the 37–62 expected NCOGCs (*P* &amp;lt; 0.001, Fisher’s exact), indicating that like COs, full-length SC is crucial for the repair of DSBs into NCOGCs. Indeed, using these data, the rate of NCOGC in an SC-deficient female can be estimated as approximately 1x10^−10^ per bp per meiosis, markedly lower than the wild-type rate of 1.9x10^−8^ NCOGCs per bp per meiosis (Hilliker *et al.* 1994; Miller *et al.* 2016b). This raises the obvious question, which will be considered next: if they are rarely repaired as COs or NCOGCs, what *is* the fate of DSBs that occur in SC-deficient flies?

### Most DSBs in SC-deficient females are repaired by an error-free process

In addition to meiotic CO or NCOGC, other potential mechanisms exist for repair of meiotic DSBs. One such mechanism, NHEJ, is often described as an error-prone process resulting in deletions (Bétermier *et al.* 2014), but data suggest the canonical NHEJ pathway is a higher-fidelity system than previously believed (Kabotyanski *et al.* 1998; Feldmann *et al.* 2000). Alternatively, single-strand annealing (SSA), alternative end-joining (alt-EJ), and microhomology-mediated end-joining (MMEJ) are pathways that may result in small deletions that could be detected as novel deletion polymorphisms in whole-genome sequencing data (Wang *et al.* 2003, 2005; Guirouilh-Barbat *et al.* 2007; Rass *et al.* 2009). Finally, repair of DSBs using the sister chromatid as a template may occur and leave little or no evidence detectable by whole-genome sequencing.

Gene conversion with the sister chromatid has been shown to be a significant repair pathway in both *S. cerevisiae* (Goldfarb and Lichten 2010) and mammalian cells (Johnson and Jasin 2000), thus it is reasonable to assume it may be active during Drosophila female meiosis as well. Indeed, ring chromosome assays have shown a decrease in the recovery of ring chromosomes in the absence of *c(3)G*, suggesting breaks in *c(3)G* homozygous females can be repaired by intersister recombination (see Discussion for a description of the ring chromosome assay) (Sandler 1965). Furthermore, in both *FM7/+*; *c(3)G^68^* and *FM7/+* females with functional SC, the recovery of *Bar* revertants through unequal exchange between sister chromatids supports the hypothesis that sister chromatid exchange does occur in flies (Hall 1977; Miller *et al.* 2016a).

To determine if DSB repair in SC-deficient females occurs by an error-prone process, novel deletion polymorphisms were identified in the three previously described classes of progeny (wild type, *c(3)G^68^*/+, and *c(3)G^68^*) plus an additional class unable to repair DSBs by crossing over. Females carrying loss-of-function mutations of the SC gene *corolla* are unable to construct full-length SC, and thus have a high rate of nondisjunction, yet still make DSBs at a rate approximately 40% of wild-type (Collins *et al.* 2014), similar to the phenotype observed in *c(3)G* loss-of-function mutations. 50 individual males and females from three females homozygous for a nonsense mutation in the SC protein *corolla* (*corolla^129^*) were sequenced. Of these 50 individuals, 11 were the result of *X* chromosome nondisjunction, with 3 *X0* males and 8 *XXY* females; 9 were triplo-4; 2 were nondisjunctional for both the *X* and *4^th^* chromosomes; and no *X* or *4^th^* chromosome mosaics were observed (Table S1). The genetic background of the *2^nd^* and *3^rd^* chromosomes of females homozygous for *corolla* was not controlled, thus candidate CO and NCOGC events could not be identified, but previous studies have shown a nearly complete absence of exchange in *corolla* homozygous females (Collins *et al.* 2014).

In all four classes of progeny (wild type, *c(3)G^68^*/+, *c(3)G^68^*, and corolla^*129*^), *de novo* deletions were searched for using two different approaches (see Methods). First, vcf files generated by SAMtools (Li *et al.* 2009) were analyzed for deletion polymorphisms (these are generally less than 20 bp), and second, larger deletions were identified using Pindel (Ye *et al.* 2009). Separately, the output of GATK HaploType caller (McKenna *et al.* 2010) was compared to SAMtools and was found to produce similar results, thus only data from SAMtools was analyzed. Both approaches identified a similar number of *de novo* deletions per fly in all four classes of progeny. Specifically, SAMtools identified 11 deletions 1–11 bp in size in previously published data from 196 wild-type males, a single 21-bp deletion in 40 *c(3)G*/+ offspring, 8 deletions ranging from 1–14 bp from 93 *c(3)G* offspring, and one 3-bp deletion from 50 *corolla* individuals (Table S6). Pindel identified only one large novel deletion among all genotypes, a complex 17-bp deletion in a *c(3)G* homozygous male.

Because DSBs are made at different rates in wild type and SC-deficient mutants, comparing the absolute number of deletions among progeny is not informative. A more accurate measure of deletion frequency is deletions per DSB. While approximately 18–20 DSBs are made during wild-type meiosis, only 4–8 are made in the SC mutants studied here (Mehrotra and McKim 2006; Collins *et al.* 2014). Although the number of DSBs made in *c(3)G^68^* heterozygous females is unknown, it is assumed to be similar to wild type because the landscape of CO and NCOGC events is similar to wild type. Using these estimates, the rate of deletions per DSB per meiosis (assuming only 25% of DSBs are recovered in the offspring) can be calculated as approximately 1% for wild-type and *c(3)G*/+ females, 3% for *corolla* females, and 10% for *c(3)G* females. The rate for *c(3)G* females is significantly higher than wild type (*P =* 0.008, Student’s 2-tailed T-test) but the rate for *corolla* females is not (*P* = 0.6, Student’s 2-tailed T-test).

Thus, while there is a subtle but significant increase in the rate of *de novo* deletions recovered in offspring from *c(3)G* mothers, this does not sufficiently explain what happens to the majority of DSBs made during meiosis. It does suggest that the majority of DSBs are repaired by a non-error-prone process between the homolog or with the sister chromatid. Of course, secondary alignment and/or analysis errors may make *de novo* deletions difficult to detect.

To test whether the analysis approach was robust enough to detect both large and small deletions, 200 *D. melanogaster* genomes with novel random single nucleotide and deletion polymorphisms were generated computationally. Two different classes of genomes were created, 100 with deletions 1–10 bp in size, and 100 with deletions 1–1,000 bp in size (Table S3). Synthetic reads were generated based on these genomes and aligned and analyzed using the same steps as the experimental samples. A total of 713 synthetic deletions were generated, with 339 1–10 bp deletions and 374 1–1,000 bp deletions. SAMtools identified 86% of 1–10 bp deletions on the *X*, *2^nd^*, and *3^rd^* chromosomes. Pindel recovered 57% of synthetic 1–1000 bp deletions (213 of the 374) with the highest fraction of deletions recovered on chromosome *2L* (72%) and the fewest on chromosome *2R* (44%) (Table S7). These models indicate that had deletions occurred at a higher rate, the additional small and large deletion polymorphisms created by error-prone repair mechanisms should have been detected in offspring from the *c(3)G* or *corolla* females.

### De novo transposable element insertions are more frequent in c(3)G^68^ homozygous females

**Figure 2:**
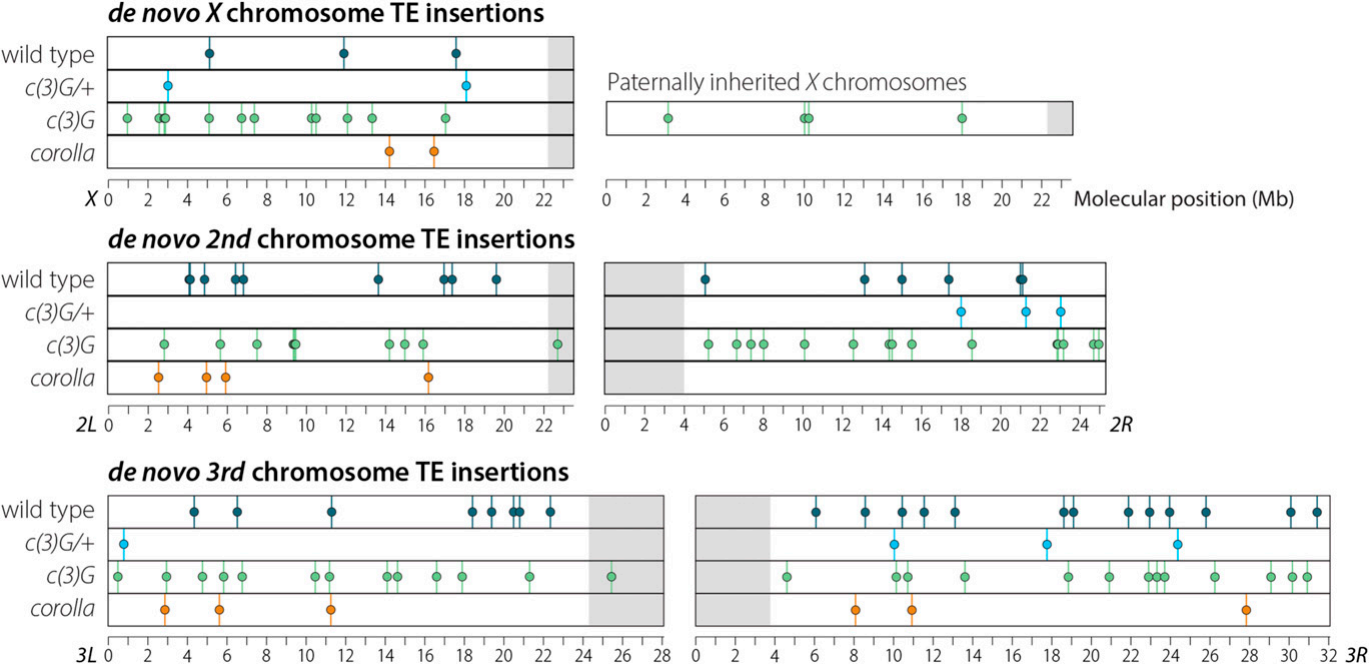
Novel TE insertions identified after a single round of meiosis for all four classes of offspring analyzed in this study. Details about insertion position and class of TE inserted can be found in Table S8.

While the SC is essential for repair of DSBs as CO and NCOGCs, it is unknown if the SC regulates other molecular events such as the movement of TEs. Absence of the yeast *c(3)G* homolog *Zip1* has been shown to result in a decreased insertion rate of the retrotransposon *Ty1*, suggesting a role for the SC in TE movement in some organisms (Dakshinamurthy *et al.* 2010). The rate at which TE movement occurs during Drosophila meiosis is unknown, therefore to determine the baseline transposition rate, previously published data were used to identify 44 novel TE insertions from the *X*, *2^nd^* and *3^rd^* chromosomes from 196 wild-type individuals (Miller *et al.* 2016b) (Figure 2, Table S8). In this dataset, a single novel insertion on the *4^th^* chromosome was observed but is not included in the rate calculations (Table S8). Seven of the 44 insertions occurred close enough to a polymorphism to confirm through linkage that they could only have been maternally inherited. For example, male cs13.13 carries a novel *roo* insertion on the *w^1118^ X* chromosome that is not seen in the 11 other male siblings that also inherited the *w^1118^* haplotype from the same female. It is not possible to definitively determine which parent the remaining 37 events were inherited from due to low SNP density. Using these data, a per arm rate of *de novo* euchromatic TE insertion can be estimated as 0.18 insertions per arm per meiosis [(44 events × 4 haploid meiotic products) / (196 meiosis × 5 arms)], meaning that while a novel euchromatic TE insertion occurs in approximately 1 in every 5 meioses, it would only be recovered in approximately 1 in every 20 progeny.

This same approach was then applied to the *X*, *2^nd^*, and *3^rd^* chromosomes from *c(3)G/+* and *c(3)G* offspring. In the 40 *c(3)G/+* offspring, 9 novel insertion events were identified—2 on the *X* chromosome, 3 on chromosome *2R*, 1 on chromosome *3L*, and 3 on chromosome *3R* (Figure 2, Table S8). Recovery of 9 novel insertion events in 40 individuals when surveying 5 chromosome arms gives a per arm *de novo* rate of transposition of 0.18 insertions per arm per meiosis, identical to the rate observed in wild type.

In *c(3)G* homozygotes, *de novo* transposition events were identified on all *2^nd^* and *3^rd^* chromosomes as well as the 68 maternally inherited *X* chromosomes from the 93 non-intersex males. Among the 440 maternally inherited chromosomes from these 93 males [68 *X* chromosomes + (93 offspring × 4 autosomes)], 64 novel transposition events were identified—12 on the *X* chromosome, 10 on *2L*, 15 on *2R*, 13 on *3L*, and 14 on *3R* (Figure 2, Table S8). Analysis of sibling *X* chromosome haplotypes confirmed that all 12 maternally inherited *X* chromosome insertions were *de novo*. Considering the 440 maternally inherited chromosomes together, the per arm per meiosis rate of novel transposon insertion events in *c(3)G* offspring was 0.58 insertions per arm per meiosis for the *X*, *2^nd^*, and *3^rd^* chromosomes, which is significantly higher than wild type (*P* &amp;lt; 0.001, Chi-square test). There was not a significant difference in the per-arm per meiosis insertion rates of 0.71, 0.54, and 0.58 for the maternally inherited *X*, *2^nd^*, and *3^rd^* chromosomes, respectively (*P* = 0.36, one-way ANOVA). The 25 paternally inherited *X* chromosomes were analyzed separately and 4 *de novo* TE insertions were observed resulting in a *de novo* transposition rate of 0.64 insertions per arm per meiosis. This rate was not statistically different from that observed for maternally inherited chromosomes (*P* = 0.58, Student’s T-test) and was also significantly higher than wild type (*P* = 0.002, Chi-square test).

To help delineate whether the increase in *de novo* transposition events is a general property of SC-deficient females or specific to *c(3)G^68^* homozygous females, novel TE insertions were identified in offspring from the previously described *corolla* mutant females. Using the same approach as above, 12 *de novo* transposon insertions were identified on the *X*, *2^nd^*, and *3^rd^* chromosomes of 50 individual offspring (Figure 2, Table S8). Of the 12 novel insertions identified, none occurred on the *X* in a male with a paternally inherited *X* chromosome, and one occurred on the *X* chromosome of an *XXY* female carrying two maternally inherited *X* chromosomes. The distribution of events was similar to that observed in wild type. These 12 events were recovered from all five chromosome arms, giving a rate of 0.19 insertions per arm per meiosis in *corolla* mutants, similar to the rate of 0.18 observed in both wild type and *c(3)G^68^/+* heterozygous females but significantly less than the observed *de novo* TE insertion rate in *c(3)G^68^* homozygous females. This suggests that the increase in *de novo* TE insertions may not be a general property of SC-deficient mutants, but may be specific to *c(3)G^68^* homozygotes.

Previous work has shown that the *de novo* movement of TEs in somatic cells contributes to cellular diversity in Drosophila (Eickbush and Eickbush 2011; Treiber and Waddell 2017). While relatively low depth of coverage and difficulty with phasing due to limited SNP density make identifying post-meiotic somatic TE insertions on the autosomes challenging, it is easier to identify mosaic insertions on the single *X* chromosome carried by males. None of the 6 *de novo X* chromosome insertions among males from wild type, *c(3)G/+*, and *corolla* females appeared to be somatic events. In *c(3)G* offspring, however, two of the 16 *de novo X* chromosome insertions identified were likely somatic events (males c3g-hom-7.14 and c3g-hom-9.7, Table S8). Both of these insertions were *Doc* elements, similar to somatic events identified by Treiber and Waddell (2017), and are present in approximately 50% of reads, suggesting they happened after the first but before the second mitotic division. It is likely that additional somatic insertions occurred in later mitotic divisions that are not detectable in this dataset given the depth of coverage in this experiment and challenges in identifying these events using short reads.

### De novo copy-number variation occurs in the absence of full-length SC

Copy-number variation is a significant source of genetic variability within populations (Kaminker *et al.* 2002; Lee and Langley 2010). CNVs may be beneficial or deleterious to an individual and may involve a large or small number of genes. Previous studies in *D. melanogaster* have revealed surprisingly high rates of *de novo* CNV both in single offspring and shared among several siblings (Watanabe *et al.* 2009; Cardoso-Moreira and Long 2010; Cardoso-Moreira *et al.* 2012; Miller *et al.* 2016b). Large CNVs have been found to frequently form between sister chromatids and are flanked by transposons, suggesting that TEs may play a key role in *de novo* CNV formation (Miller *et al.* 2016b). Smaller CNVs that average 50–100 bp have also been identified in Drosophila, and these are associated with replication timing, but not with TEs (Cardoso-Moreira *et al.* 2011). It is not known whether functional SC is critical for CNV formation either between sisters or homologs.

**Figure 3:**
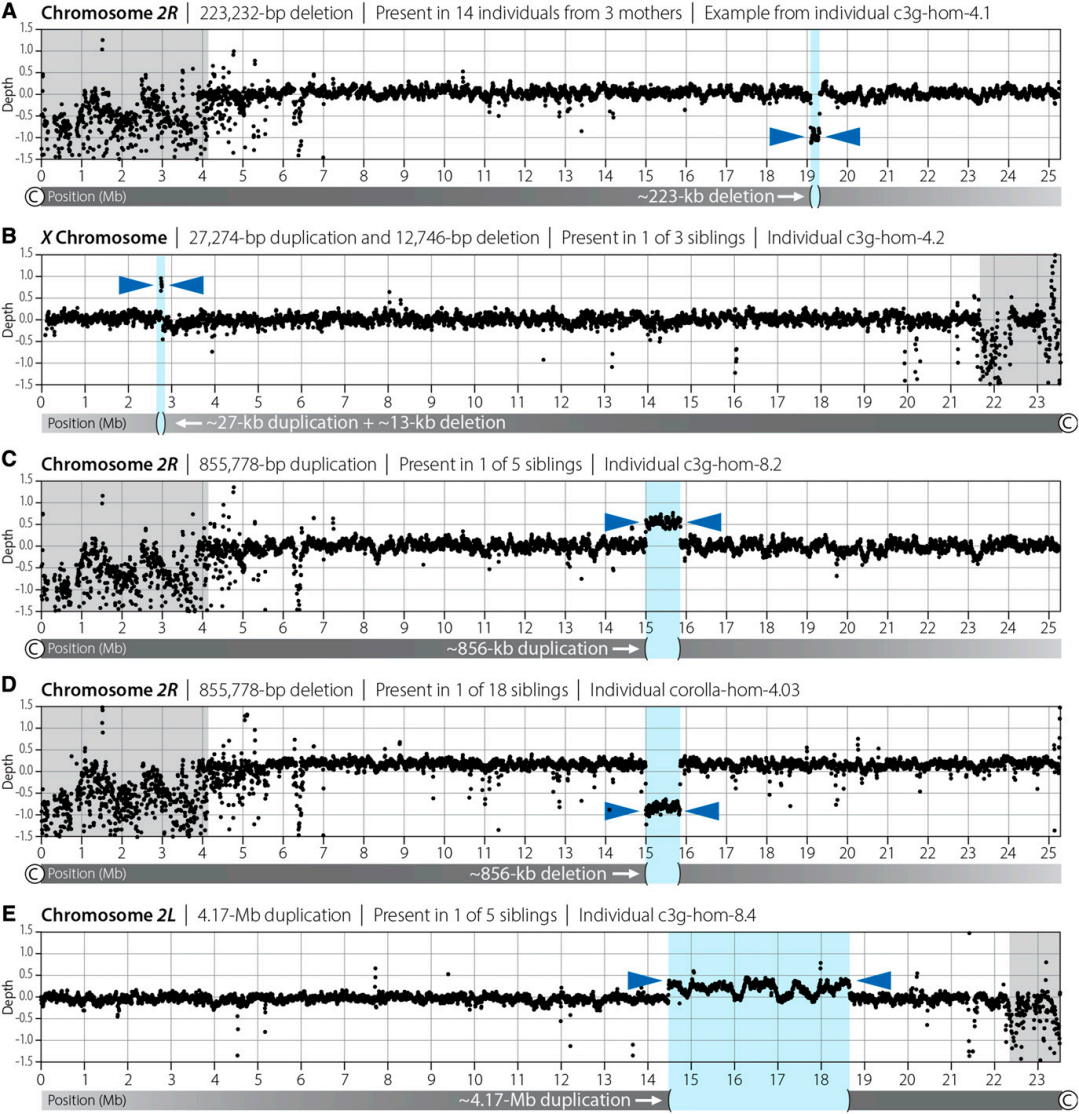
Copy-number variants recovered in this study (details are in Table S9). Gray shading indicates heterochromatic regions and © represents the centromere. (A) 223-kb deletion shared among 14 males from 3 different females that likely occurred at least two generations prior. (B) A complex 27-kb duplication and 13-kb deletion at the *white* locus that was recovered in a single offspring and is likely to be *de novo* based on sibling haplotypes lacking the rearrangement. (C, D) An 856-kb duplication identified in a single male from a *c(3)G^68^* female has identical start and end coordinates as an 856-kb deletion recovered in a single male from a corolla^*129*^ female and is identical to an 856-kb duplication recovered in a single male from a wild-type female from a prior study (Miller *et al.* 2016b). (E) A large 4.17-Mb duplication observed in a single individual that is likely mosaic based on its lower log_2_ ratio of 0.25.

Here, large CNVs were identified by plotting depth of coverage for all chromosome arms for all individuals from *c(3)G^68^/+*, *c(3)G^68^*, or corolla^*129*^ females and revealed 5 total events. No *de novo* CNV events were observed in offspring from *c(3)G^68^*/+ females, 4 events were recovered in offspring from *c(3)G^68^* females, and 1 event was seen in an offspring of a corolla^*129*^ female (Figure 3, Table S9). Among the 4 events recovered from the *c(3)G^68^* females, one was shared among multiple siblings—a 223-kb deletion of chromosome *2R* involving 27 genes, which was recovered from 14 male offspring from females #4, 5, and 6 (Figure 3A). That this event was observed in individuals from multiple crosses of individual males and females makes it likely that the deletion occurred at least two generations prior and did not have a significant impact on the reproductive fitness of those individuals carrying it.

The remaining CNVs recovered were only observed in single individuals and, based on haplotype analysis, were likely *de novo* events. One event, a complex *de novo* CNV involving both a deletion and a duplication was identified at the *white* (*w*) locus on chromosome *X* in one male from a *c(3)G^68^* homozygous female (Figure 3B). This event was mediated by unequal crossing over between *roo* elements. Previous work describing ectopic recombination in *D. melanogaster* focused on unequal exchange between *roo* elements at the *w* locus, similar to the event observed here (Goldberg *et al.* 1983).

Two *de novo* CNVs (one duplication and one deletion) occurred at the exact same position on chromosome *2R* in two individuals. The duplication was recovered from the offspring of a *c(3)G^68^* female, while the deletion was recovered from the offspring of a corolla^*129*^ female (Figure 3C,D). This 856-kb event includes 107 genes and is flanked on both sides by a *hobo* element. Remarkably, a CNV with the exact same breakpoints was identified in a previous study of individuals from wild-type females (Miller *et al.* 2016b). All three of these CNVs were *de novo* events validated using the haplotypes of the siblings that did not carry the CNV. All three crosses were between individual males and females and multiple genetic backgrounds are involved (*w^1118^*, Canton-S, and the undefined corolla^*129*^ background), thus these CNVs are not variants segregating at low frequency in the population and are recurrent *de novo* events.

The final CNV observed, a 4.2-Mb duplication on chromosome *2L* not flanked by a TE or low-complexity sequence, was recovered in a single male from a *c(3)G^68^* female (Figure 3E). Analysis of read pairs revealed that this was a tandem duplication, with reads mapping to the proximal end of the duplication linked to reads mapping to the distal end of the duplication. The log_2_ depth-of-coverage ratio for this interval is 1.25, 0.25 higher than expected for a diploid and less than the log_2_ depth-of-coverage ratio of 1.5 that would be seen in an autosomal duplication occurring before the first mitotic division. Thus, this duplication is present in half the cells in the individual sequenced and likely occurred during the first mitotic division, possibly as a consequence of a re-replication event that was then repaired by recombination between the duplicated segments (Green *et al.* 2010). It is notable that the fly was able to tolerate such a large duplication, involving 513 genes, present in half of all cells. Although the possibility that there was selection against cells carrying the large duplication cannot be excluded, a log_2_ depth-of-coverage ratio of 1.25 does strongly suggest there was limited selection against those cells with the duplication. If selection was acting strongly on these cells the log_2_ ratio would fall below 1.25 and perhaps become undetectable.

## Discussion

In this study, whole-genome sequencing was used to evaluate individual offspring from females either heterozygous or homozygous for loss-of-function alleles of two different SC components, *c(3)G* and *corolla*. Loss-of-function variants in either gene prevent the construction of full-length SC and reduce the number of meiotic DSBs to approximately 20% of wild type in *c(3)G* and 40% of wild type in *corolla* (Mehrotra and McKim 2006; Collins *et al.* 2014). Previous studies have shown that these DSBs are rarely repaired as COs (Gowen 1933; Hall 1972; Collins *et al.* 2014), therefore how DSBs are repaired in the absence of SC remains an open question.

While evaluating the results of this study, several possible confounding factors should be kept in mind. First, as not all experiments were done at the same time, subtle differences in food, humidity, or temperature could affect these results. Next, the number of females from which offspring were collected from was not balanced. For example, while 96 individuals were taken from 10 *c(3)G* females, 40 males were taken from 2 *c(3)G**/+* females. This could result in subtle female-specific effects being amplified in the results. Finally, offspring for sequencing were collected between days 12 and 15 post-fertilization and flies which would have eclosed after day 15 were not collected. Previous work has shown that maternal age affects CO events (Hunter *et al.* 2016), and it is not unreasonable to assume that age may have an impact on other factors such as the formation of CNVs and movement of TEs.

CO and NCOGC events were identified in males from *c(3)G^68^*/+ females and compared to wild type (Figure 1). In agreement with previous studies of crossing over in *c(3)G^68^*/+ (Hall 1972), the frequency and distribution of events identified in this study appears similar to wild type, although the small sample size may mask subtle differences in the heterozygous genotype. Next, among 93 males from *c(3)G^68^* homozygous females, no CO events and a single NCOGC event were identified. Recovering no CO events was not unexpected based on prior studies of *c(3)G* homozygous females (Gowen 1933; Hall 1972). The single NCOGC recovered was defined by two polymorphisms and validated using PCR and Sanger sequencing. While this event is likely real and not an artifact of sequencing or alignment, it is not possible to definitively determine whether this event occurred during meiosis or was instead a pre-meiotic conversion event.

To determine the fate of DSBs made in females unable to construct full-length SC, novel deletion polymorphisms were identified in wild type, *c(3)G^68^*/+, *c(3)G^68^*, and corolla^*129*^ offspring. Comparing the absolute number of deletions between genotypes is not accurate as DSBs in SC mutants are made at a fraction of the wild-type rate, thus a better comparator is deletions per DSB. When analyzed in this way, approximately 10% of DSBs made in individuals from *c(3)G^68^* homozygous females resulted in a novel deletion—significantly more than the 1% observed in the wild-type and *c(3)G^68^*/*+* datasets—while only 3% of DSBs in *corolla* homozygous females were recovered as deletions, an observation not different from wild type.

The increased frequency of *de novo* deletions in offspring from *c(3)G^68^* homozygous females does not explain the fate of the remaining 90% of DSBs made in these flies. Sandler (1965) used a ring chromosome assay in a *c(3)G* background to determine whether these DSBs are repaired by sister chromatid exchange. In that assay, females carrying one ring *X* chromosome and one normal rod *X* chromosome are constructed. The absence of any exchange yields an equal number of progeny with a ring or a rod chromosome. If exchange occurs between homologs (between the ring and the rod chromosome), single CO events will result in acentric and dicentric chromosomes that will not segregate properly, and because both chromosomes will be equally affected, an equal but decreased number of ring and rod chromosomes will be recovered. In the absence of homologous recombination but in the presence of sister chromatid recombination, rod chromosomes will be transmitted at a normal level, since sister chromatid exchange events on a rod chromosome will not affect their structure or segregation. Single sister chromatid exchange events on a ring chromosome will result in a large dicentric ring that will not segregate properly, thus the number of progeny inheriting a ring will be lower than the number of rod chromosomes recovered.

Sandler found that in the *c(3)G* mutant background, rings are recovered less frequently than the homologous rod *X* chromosome, demonstrating that sister chromatid exchange is occurring in the absence of C(3)G (Sandler 1965). Separately, Hall (1977) was able to recover *Bar* revertants from *FM7/+*; *c(3)G^68^* females which, in the absence of homologous recombination, occur through unequal exchange between sister chromatids (Hall 1977). Thus, taken together, it is most likely that the majority of DSBs in SC-deficient flies are repaired either by a high-fidelity repair process such as canonical NHEJ or by sister chromatid repair.

These results also help clarify the role the SC plays in determining which chromatid is used for DSB repair. During mitosis, an intersister bias exists as the sister chromatid is preferred for repair. Alternatively, there is interhomolog bias during meiosis, as the homolog is preferred for DSB repair. How the cell determines which chromatid to use for repair is an area of active research. In *S. cerevisiae*, DMC1 (the *E. coli* RecA homolog) promotes interhomolog DSB repair and Dmc1-deficient yeast only undergo intersister DSB repair during meiosis (Bishop *et al.* 1992; Schwacha and Kleckner 1997). Both *D. melanogaster* and *C. elegans* lack an ortholog of DMC1, thus it is unclear how interhomolog bias is established during meiosis. One hypothesis is that these species no longer need DMC1 because the SC is built prior to the initiation of recombination. The data presented here, along with prior studies showing that sister chromatid repair occurs in the absence of SC, support the hypothesis that the SC plays a critical role in establishing interhomolog bias in Drosophila and that in the absence of SC, repair from the sister chromatid is possible.

Whole-genome sequencing of individual meiotic products allowed for the characterization of other meiotic events as well, such as whether the absence of full-length SC affects the movement of TEs. The observed rate of novel TE insertions in this study was significantly higher in offspring from *c(3)G^68^* females when compared to the other three classes of progeny studied (wild type, *c(3)G^68^/+*, and corolla^*129*^). Surprisingly, the elevated rate in maternally derived *c(3)G^68^* chromosomes was similar to the rate from paternally derived *X* chromosomes, which came from males with two wild-type copies of *c(3)G*. The increased rate of *de novo* transposition in an SC mutant may provide new clues as to other roles that SC components might play in facilitating or preventing the movement of TEs.

This increased rate of novel TE insertion in *c(3)G^68^* mutants could be explained by a model in which C(3)G prevents mobilized TEs from inserting into genomic DNA. In the absence of C(3)G, a greater number of TEs may be available to insert into nuclear DNA. A higher number of active TEs may also explain why the rate of TE insertions was similar on *X* chromosomes derived from wild-type males, but this would require that TE insertions occur post-fertilization.

Alternatively, the increased rate of TE insertion in *c(3)G* females could be explained by differences in genetic background leading to an increased rate of transposition (Kidwell *et al.* 1977). Previous studies have reported “bursts” of TE insertions from a specific TE class and attributed the observation to differences in genetic background (Pasyukova and Nuzhdin 1993; Page *et al.* 2007; Guerreiro 2011). It is worth noting that 20 of the novel insertions identified in the homozygous *c(3)G^68^* dataset were *doc* elements, which seems to support the idea that different genetic backgrounds might exhibit different rates of transposition. Although, the genetic background in these experiments was somewhat controlled as females both heterozygous and homozygous for *c(3)G^68^* were heterozygous for the same *w^1118^* and Canton-S *X* and *2^nd^* chromosomes, which were from the same stocks used in the wild-type experiment (Miller *et al.* 2016b). These two stocks differed in that females heterozygous for *c(3)G^68^* carried one copy of a *w^1118^ 3^rd^* chromosome, while those homozygous for *c(3)G^68^* did not. Although the fact that these chromosomes have been used in previous experiments might reduce the contribution of genetic background to the elevated TE insertion rate, it may not completely eliminate it. The background of *corolla* mutants was not controlled. A unifying explanation may be that *c(3)G* itself plays a previously unappreciated role in the prevention of TE movement and that this is separate from the role, if any, played by fully functional SC.

Analysis of reads spanning *de novo* TE insertion sites revealed that two of the 16 total *de novo* TE insertions identified on the *X* chromosome were mosaic. Approximately 50% of reads at the TE insertion site spanned the junction and did not contain TE sequence, suggesting that the insertion occurred after the first, but before the second mitotic cell division. Both of the likely mosaic insertions were *doc* elements, with one occurring on a maternally inherited *X* chromosome and the other on a paternally inherited *X*. Removing both of the insertions does not significantly alter the overall rate of TE insertions per arm per meiosis calculated using the observed data, but does decrease the rate of insertions observed on the paternally inherited *X* chromosome from 0.64 to 0.48, suggesting a *de novo* insertion on the paternal *X* chromosome would be recovered approximately 1 in every 8 meioses instead of 1 in every 6.

The increased rate of TE movement in *c(3)G* homozygotes suggests that SC components may play a role in mediating TE movement. While the tripartite structure of the SC is widely conserved across species, the amino acid sequence of the components themselves are not (Handel and Schimenti 2010; Fraune *et al.* 2012, 2013). Indeed, the rate of the amino acid change of SC components makes it challenging to find homologs of the transverse filament protein *c(3)G* and other previously identified *D. melanogaster* SC components even in other species of Drosophila (Hemmer and Blumenstiel 2016). At a larger evolutionary scale, there is little sequence homology among lateral element, transverse filament, or central element components of *C. elegans*, *D. melanogaster*, and *M. musculus*, suggesting the rapid rate of evolution is not limited to the Drosophila species group (Fraune *et al.* 2016). This is unlike components of the recombination machinery, which associate with the SC during meiosis and are highly conserved at the amino acid level (Zalevsky *et al.* 1999; Villeneuve and Hillers 2001). Thus, it is reasonable to posit that the rapid rate of evolution observed for SC components across species and organisms may in part be a response to the ongoing evolution of TEs.

The recovery of large TE-mediated CNVs in females unable to construct SC demonstrates that these types of CNVs can occur independent of normal meiotic synapsis and DSB formation, perhaps depending only on the presence of a chromosome axis. Of course, it is also possible the events recovered here, including the events identified in the wild-type dataset, occurred during mitosis. While this study only sought to identify large CNVs, it is likely that many smaller CNVs occurred which were not identified. Indeed, previous studies in Drosophila using both microarray and whole-genome sequencing have shown that both small and large CNVs are segregating in wild populations (Emerson *et al.* 2008; Cridland and Thornton 2010; Cardoso-Moreira *et al.* 2011, 2012), however these studies did not evaluate the frequency of TE-mediated events because they were done using microarrays or filtered reads that partially mapped to TE sequences.

That two different TE-mediated CNV events (a deletion and a duplication) in two different genetic backgrounds were recovered at the exact same coordinates as a duplication observed in wild-type individuals was surprising and suggests that the rate of CNV formation and the ability to tolerate a large CNV may not be uniform across the genome. As would be expected in mutants with defective homologous chromosome pairing, all four TE-mediated CNV events recovered in this study appear, based on allele frequency and TE positioning, to be events between sister chromatids and not between homologous chromosomes.

Overall, this work demonstrates the wide range of insights that can be gained by analysis of individual meiotic products from both wild-type and mutant backgrounds. From this work, additional testable questions arise. For example, what happens to DSBs in SC-deficient backgrounds in the absence of the NHEJ machinery? And is an increased rate of TE movement observed in Drosophila SC mutants other than the transverse filament protein C(3)G, such as C(2)M or Cona? If it is simply the result of a deficiency of C(3)G, then the question arises of whether homologs of C(3)G, such as ZIP1 and SYCP1, also play a role in mediating TE movement during meiosis and through what mechanism. Future work may address these questions at a larger scale with additional detail as the cost of sequencing continues to fall and tools and techniques for analyzing large datasets such as this improve.
